# The development of PAT‐HD: A co‐designed tool to promote physical activity in people with Huntington’s disease

**DOI:** 10.1111/hex.13210

**Published:** 2021-02-13

**Authors:** Una Jones, Katy Hamana, Fran O’Hara, Monica Busse

**Affiliations:** ^1^ School of Healthcare Sciences Cardiff University Heath Park Cardiff UK; ^2^ Scarlet Design Tec Marina Terra Nova Way Penarth UK; ^3^ Centre for Trials Research Cardiff University Neuadd Merionnydd Heath Park Cardiff UK

**Keywords:** acceptability, co‐design, Huntington's disease, physical activity

## Abstract

**Background:**

Huntington's disease (HD) is a genetic condition resulting in movement, behavioural and cognitive impairments. People with HD have low levels of physical activity which may be compounded by insufficient support from health‐care professionals.

**Objective:**

To evaluate the initial acceptability of a co‐designed tool used within a HD clinic to promote physical activity.

**Design:**

Co‐design of a physical activity tool; acceptability evaluation.

**Setting and participants:**

Co‐design included people with HD and health‐care professionals. Acceptability was evaluated in a HD clinic in the UK.

**Main variables studied:**

A physical activity tool was co‐designed and used within a HD clinic.

**Main outcome measure:**

Acceptability as assessed by semi‐structured interviews with members of the HD clinic.

**Results:**

Forty people visited the HD clinic; 19 were given physical activity advice. Themes around who, where and how promotion of physical activity could take place were identified; concepts of benefits and barriers were threads through each theme.

**Discussion:**

We describe for the first time the co‐design of a HD specific physical activity tool. Our associated acceptability study emphasizes the importance of individualized planning of physical activities in complex neurodegenerative conditions. Perceived barriers were time and lack of knowledge of local resources.

**Conclusions:**

A simple tool can support conversations about physical activity with people with HD and is an aid to individualized goal setting. Exploring the use of PAT‐HD within a community setting and development of support systems for health‐care professionals and support workers who are in regular contact with people with HD is required.

## 1 INTRODUCTION

Huntington's disease (HD) is a single gene neurodegenerative disease resulting in movement, behavioural and cognitive impairments with death occurring, on average, 18 years after onset of motor symptoms in midlife.^1^ HD is rare, with a prevalence ranging between 12.3/100 000 and 17.27/100 000 across Europe, the Americas, Asia and Oceania^2^ The movement problems of chorea (abnormal involuntary movements), bradykinesia (slowing of movement) and dystonia (involuntary muscle contractions) begin in midlife, can co‐exist and change through the progression of the disease.^3^ These problems are compounded by slowing of information processing, depression and apathy^4^ and lead to decreasing functional independence.^5^ The complexity of the devastating psychosocial and physical consequences of HD underpin individualized approaches to care, particularly in relation to maintaining physical activity.^6^ Despite growing evidence, there appears to be a lack of uptake of exercise and/or physical activities in people with HD. Low levels of physical activity have been identified,^7^ and people with HD struggle to regularly take part in physical activity.^8^ This lack of uptake of physical activity may be compounded by difficulty accessing support from health‐care professionals.

Exercise studies have taken place at a number of locations including home‐based using an exercise DVD,^9^ a community gym,^10^ and the choice of exercising on a stationary bike at home or local gym.^11^ Although the use of an exercise DVD at home and exercising at a community gym was found to be safe, only the more intensive progressive exercise routine using the stationary bike was found to increase fitness. A multi‐centre trial with a social comparator utilized the exercise DVD with a physical activity workbook and coaching and found an increase in both self‐efficacy for exercise and self‐reported physical activity levels.^8^

These clinical trials provide rigorous evidence for effectiveness of exercise and physical activity interventions that has been integrated within recent clinical guidelines for physical therapy.^12^

The complexity of the movement, behavioural and cognitive dysfunction caused by HD and the associated slow decline in functional ability means that individuals experience different issues across their lifespan. Promotion of physical activity must therefore be individualized and adapted over time. People with HD are motivated by the potential benefits of taking part in physical activity, but the physical activity behaviour requires increasing collaboration over the time span of the condition, for example caregivers providing more input and support across the disease timespan.^6^

Promotion of exercise in the general population is challenging due to time constraints, perceived lack of patient engagement, health‐care professionals’ lack of formal education regarding physical activity and health‐care professionals’ competing priorities, for example other health promotion activities.^13^, ^14^ Physiotherapists, who are perceived as being ideally placed to promote physical activity,^15^ appear to not actually fulfil this role. Williams et al^16^ and Lowe et al^17^ acknowledge that perceived lack of both knowledge regarding physical activity and skills with which to promote change may be responsible for physiotherapists focusing on rehabilitation rather than physical activity promotion in people's day‐to‐day living.

As HD is a rare condition, health‐care professionals in general may have limited knowledge about the condition and therefore the role of personalized physical activity plans within a specialist HD clinic needs to be explored. In order to facilitate implementation and effectiveness of interventions that support long‐term behavioural change in relation to physical activity, the interventions must be developed by a range of stakeholders so as to be ecologically valid.^18^ This aligns with the principles of co‐production described by Osborne et al^19^ who suggest that service users should be involved in aspects such as design, delivery and/or evaluation of service provision. Specifically, co‐design is identified as an approach to co‐create value within a service that actively involves stakeholders to help ensure that the outcome is fit for purpose and meets their needs.^20^

The aims of this study were to co‐design an intervention to support health‐care professionals promote physical activity with people with HD and to assess that intervention for acceptability within a specific Huntington's disease clinic. As such, the study comprised three phases: Phase 1, development of a prototype physical activity tool; Phase 2, co‐design of the physical activity tool and Phase 3, acceptability of the physical activity tool with health‐care professionals working in HD.

Ethical approval was provided by the School of Healthcare Sciences, Cardiff University for phases 1 and 2 (10/10/17) and by the Health Research Authority and Health and Care Research Wales for phase 3 (REC reference 18/HRA/2112).

## 2 MATERIALS AND METHODS

### 2.1 Phase 1: Development of a prototype physical activity tool

The aim of this phase was to develop a prototype physical activity tool utilizing a visual led methodology and synthesis of emerging evidence from previous studies. The research team worked with a co‐production community interest company, Scarlet Design (https://www.scarletdesign.com/) with expertise in visual led methodologies. Emerging evidence from exercise studies in HD ^6^, ^8^, ^11^, ^21^ and guidance from the American Society of Sports Medicine on Frequency, Intensity, Time, Type (FITT) Principle^22^ was discussed with the design company to produce a prototype physical activity tool. Additionally, twelve scenarios based on hypothetical people with HD were developed by the research team and reviewed by people with HD for use in Phase 2.

### 2.2 Phase 2*:* Co‐design of a tool to promote physical activity for people with HD

The aim of this phase was to develop a tool to promote physical activity in people with Huntington's disease for use in a clinical setting, using a co‐design approach. Relevant stakeholders were identified as people with HD and people who would support people with HD to use the tool. People with HD, n = 8, and their carers/families, n = 10, were recruited via the Huntington's Disease Association of England and Wales (HDA). Specialist HD advisors, n = 4, were recruited from the HDA. Health‐care professionals, for example medical doctor, physiotherapist, nurse working with people with HD, n = 6, were recruited via the European Huntington's Disease Network. Health‐care professionals were asked to consider being a participant in co‐design of the tool and further to consider evaluating acceptability of the tool in their HD clinic (based in the UK).

A co‐design event planned around three structured activities was facilitated by Scarlet Design. In activity 1, attendees worked together to compile a list of activities that they considered suitable for people with HD. In activity 2, the groups used ideas generated in activity 1 to hypothetically guide a person with HD to be more active. Participants used the prototype physical activity tool with the twelve scenarios that had been developed in Phase 1 for this activity. Activity 3 was focused on gaining feedback on the prototype tool.

Large scale table visual templates were used for activities that provided participants an accessible way to record their conversations and ideas. This method also allowed people to describe their ideas and for others to record them. As the information was recorded visually, participants could read and see responses of the group over the time period of the activities and therefore were free to move away from the table and return when necessary.

Ideas generated and data collected during activities were used to co‐design the physical activity tool for people with HD (PAT‐HD) alongside development of a user guide to support health‐care professionals. The user guide provided a background to the development of PAT‐HD and twelve completed PAT‐HD for fictional people with HD from across the spectrum of the condition. Key elements of feedback from participants at the event on the prototype tool from activity 3 were that the language needed to be more person centred and that a less clinical approach should be taken. A version of PAT‐HD was then presented to and reviewed at a HDA family day attended by 104 members of the HD community. Feedback at this event was the need to provide information on why physical activity is beneficial and to have a simple approach. Synthesis of ideas generated at the co‐design event, feedback from the co‐design event and the family day resulted in the production of the physical activity tool PAT‐HD for use in Phase 3.

### 2.3 Acceptability of the physical activity tool with health‐care professionals working in HD

The aim of this phase was to explore the acceptability of using PAT‐HD within a HD specific clinic, by the health‐care professionals using the tool. Three UK clinics expressed an interest in the study, and one clinic agreed to participate.

The HD clinic used PAT‐HD and associated user guide over a nine‐month period. The number of people visiting the clinic and data related to physical activity was collected in accordance with The Royal Society for Public Health Impact Pathway—Physical Activity.^23^ This pathway records the following: how many times physical activity was raised with an individual, how many times physical activity advice was offered to an individual, the advice provided, number of referrals to an exercise referral scheme and the number of people who did not want to discuss physical activity.

Individual semi‐structured interviews were carried out at the end of the nine‐month study period with three members of the HD clinic team who had opportunity to discuss physical activity: a research physiotherapist, a HD nurse specialist and a medical consultant. Interviews were carried out in person at the clinic, lasted between 20 and 35 minutes and were audio‐recorded. An interview schedule was developed based on previous literature relating to physical activity and HD^6^, ^9^ and the normalization process theory for implementing change.^24^ Interview questions included topics such as: the participant's familiarity of use of PAT‐HD; any changes to practice as a consequence of using PAT‐HD; barriers and enablers to promotion of physical activity in HD; changes that could be made to PAT‐HD; alternative methods of promotion of physical activity in HD.

Data related to clinic visits were analysed descriptively. Data from the interviews were transcribed verbatim. Key themes and salient concepts were identified through inductive thematic analysis^25^ and carried out independently by two authors. Initial coding followed familiarization of the data with codes being generated by selecting segments of text and assigning to organize the data into meaningful groups. Credibility of the findings was promoted by double coding and awareness of one's own biases.^26^ Themes and subthemes were identified through reflexive discussions between the two researchers.

## 3 RESULTS

### 3.1 Phase 1: Development of a prototype physical activity tool

**FIGURE 1:**
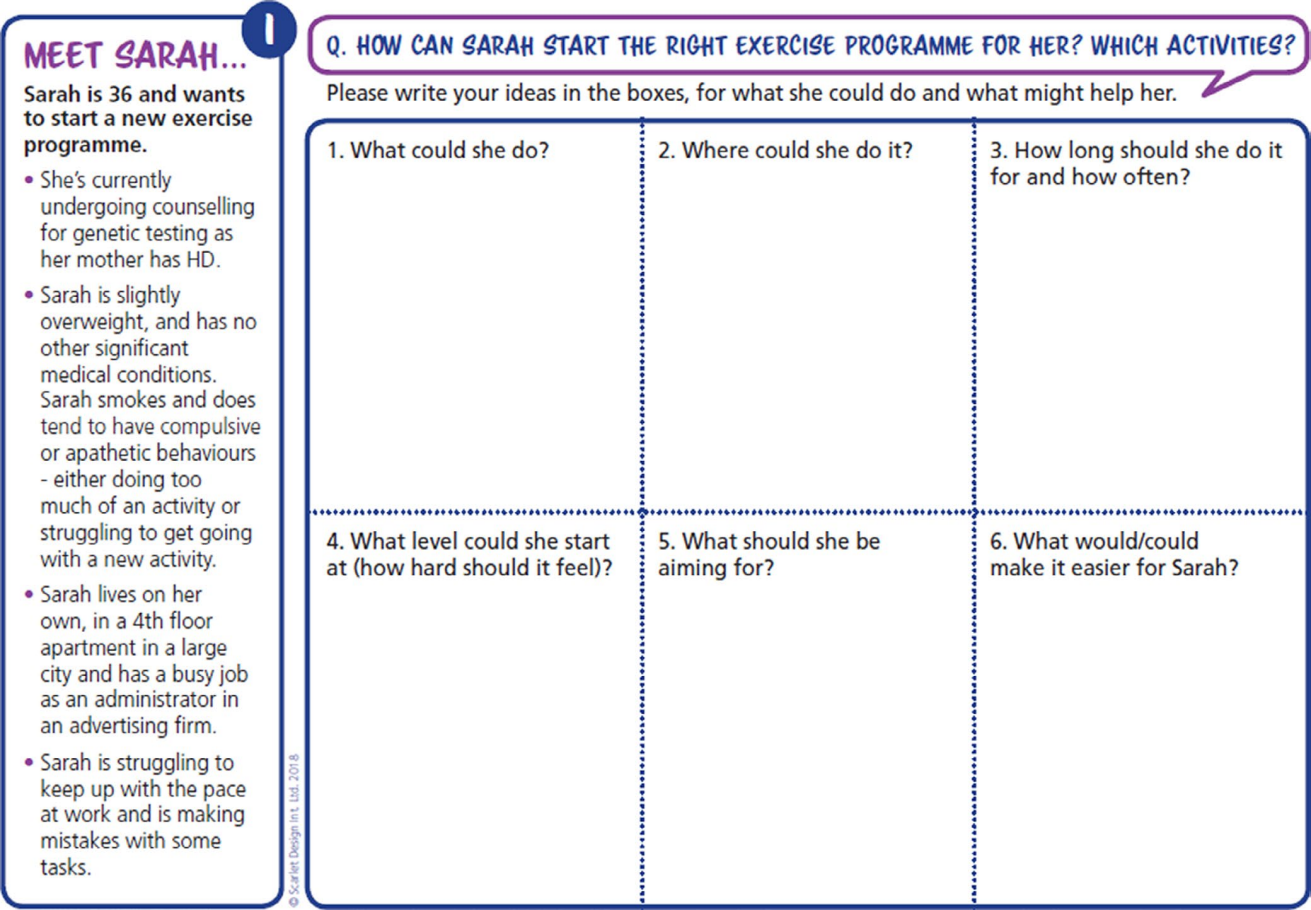
Prototype physical activity tool

The prototype tool developed, see Figure 1, was a single‐sided A4 sheet of paper with details of a hypothetical person with HD and six boxes each with a question related to the development of a physical activity plan. The structure of the six boxes aimed to promote planning of physical activity. Questions in the boxes were developed using the FITT principles: What could he/she do? (Type); Where could he/she do it?; How long should he/she do it for and for how often? (Timing and Frequency); What level could he/she start at (how hard should it feel) (Intensity)?; What should he/she be aiming for?; What would/could make it easier for him/her?

### 3.2 Phase 2*:* Co‐design of a tool to promote physical activity for people with HD

**FIGURE 2:**
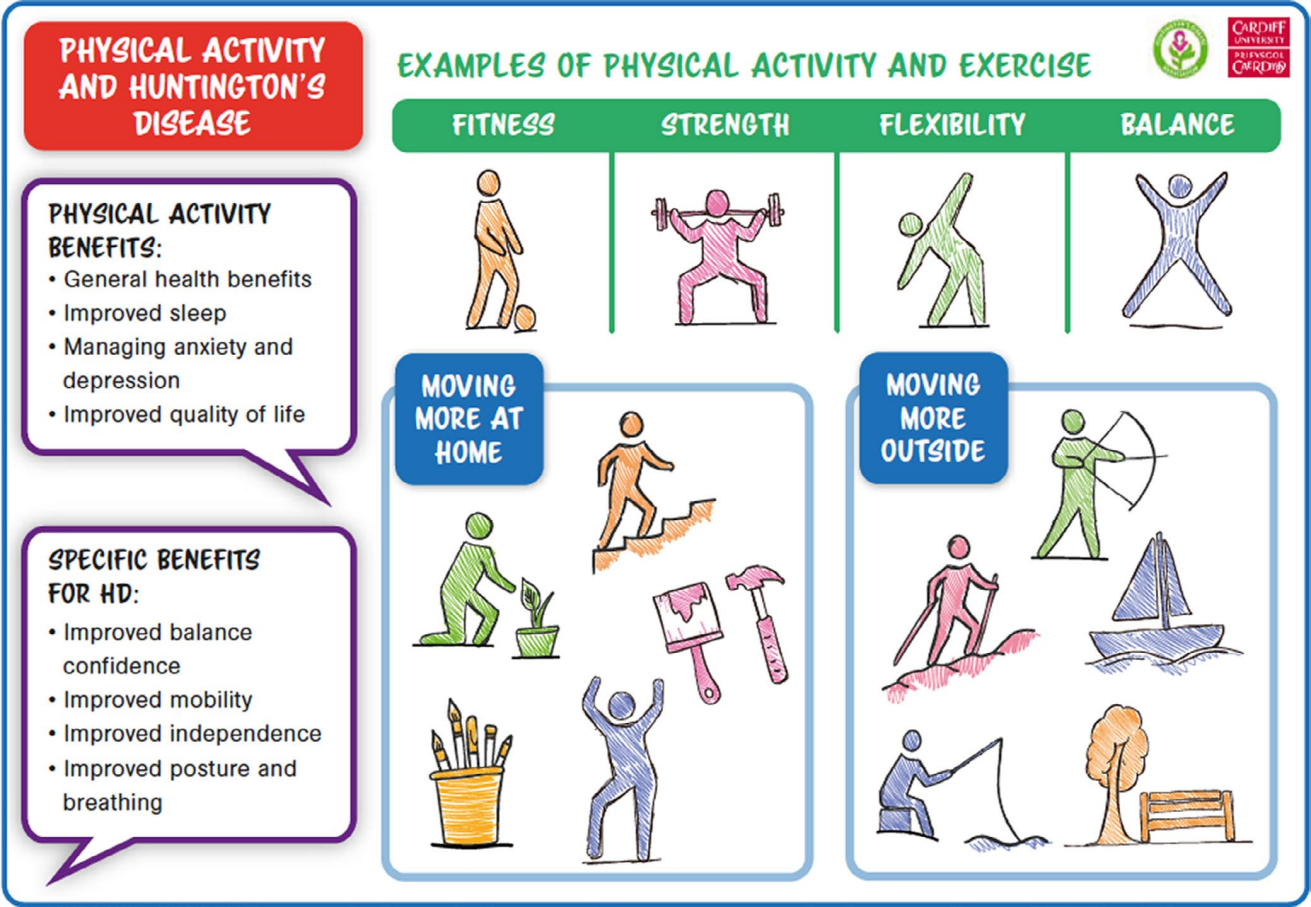
Front of physical activity tool for people with HD (PAT‐HD)

PAT‐HD was co‐designed as an A4, two‐sided piece of paper. The front page, see Figure 2, is designed to facilitate a conversation between a health‐care professional and a person with HD about physical activity including the general benefits, based on the World Health Organization guidance^27^ and benefits specific to HD based on a recent systematic review.^21^ Activities suggested by participants in activity 1 of the co‐design event were grouped in categories of fitness, strength, flexibility and balance as key components of physical fitness.^22^ Illustrations of activities, created by the design agency, were used for visual impact and were categorized as moving more at home and moving more outside to capture all stages of the condition.

**FIGURE 3:**
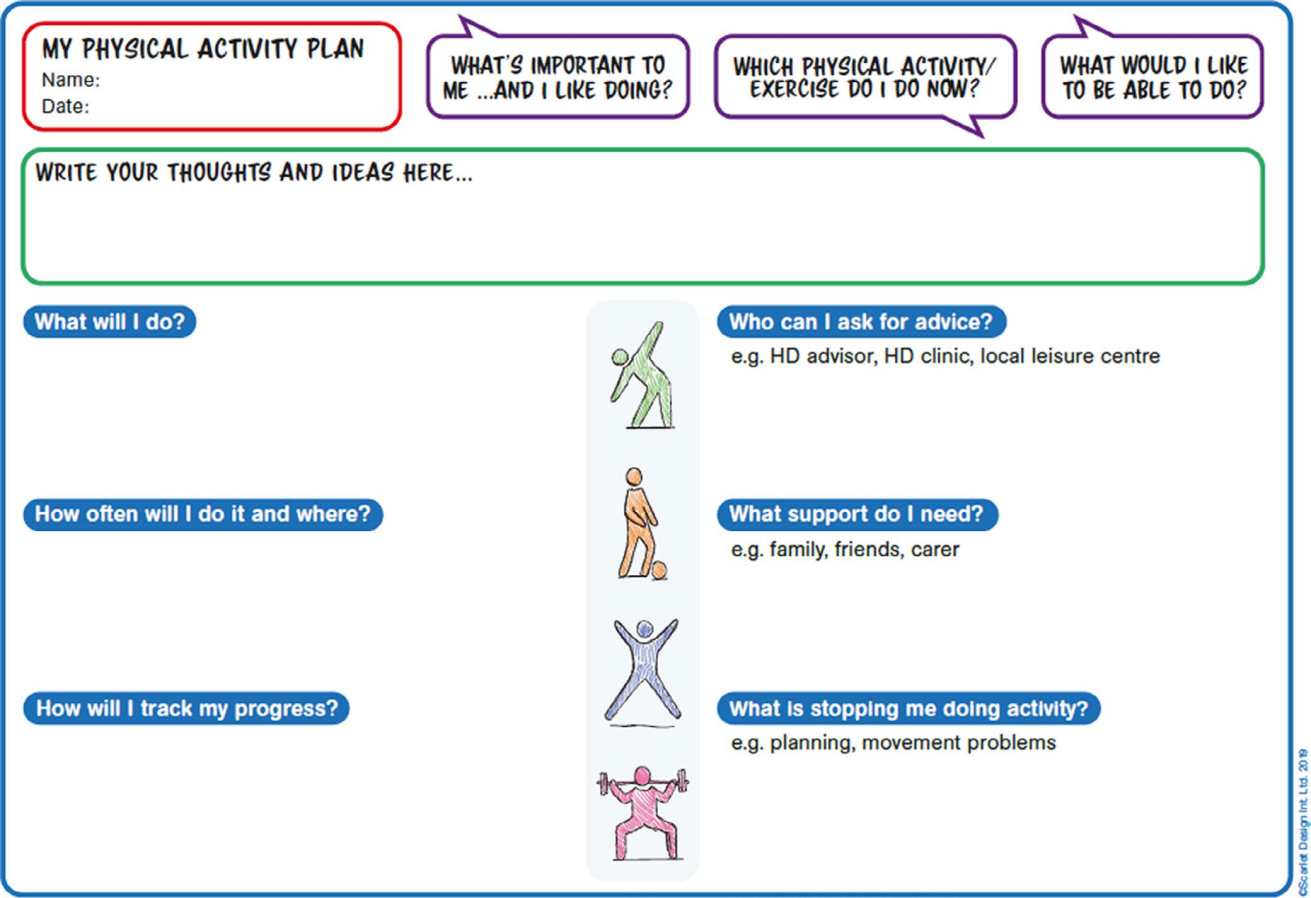
Reverse of PAT‐HD

The reverse page, see Figure 3, was designed to allow people to write a personalized physical activity plan. The plan included the following: Name and date; what is important to me, which physical activity/exercise do I do now, what would I like to be able to do; what will I do; how often will I do it and where; how will I track my progress; who can I ask for advice; what support do I need; what is stopping me from doing activity. The last three sections had prompts, for example Who can I ask for advice had prompts of HD advisor, HD clinic, local leisure centre.

### 3.3 Phase 3 acceptability of the physical activity tool with health‐care professionals working in HD

A total of 40 people with HD visited the participating HD clinic during the 9 months. Physical activity was discussed with 21 individuals with 19 people being offered advice. Advice included walking (n = 6), getting out and about (n = 4), keep active (n = 4), yoga (n = 2), pilates (n = 1) and continue to stay active (n = 4); no referrals were made to exercise referral schemes. Although no individuals declined to talk about physical activity, health‐care professionals tended to not raise the topic for discussion with those who were at an advanced stage of condition or those with severe mental health issues. There were also instances when they did not have sufficient time to discuss regular physical activity given the other competing requirements of the consultation.

Individual semi‐structured interviews were conducted with three health‐care professionals from the clinic: one research physiotherapist, one HD nurse specialist and one HD medical consultant. Three key themes and their subthemes were identified through inductive thematic analysis conducted by two researchers. These were as follows: *People* (three subthemes); *Setting* (two subthemes) and *Approach* (two subthemes). A crosscutting theme of benefits and barriers was intertwined with each main theme and will be reported within the themes.

The theme of *People* refers to both the individuals promoting physical activity and people with HD with whom physical activity is being promoted. The subtheme of professional role was voiced by all participants and expressed in relation to different health‐care professionals taking on roles that they identified as being within their specific profession's scope of practice, with physical activity promotion being perceived to be mainly the responsibility of the physiotherapist.

Medical consultant ‘I think they’d be surprised if I started spouting forth about … physical exercise …So I think having a dedicated physiotherapist to pick it up’

When promotion of physical activity in the community was discussed, HD advisors were mentioned as potentially well‐placed individuals to promote physical activity.

Nurse ‘HD advisors …go into care homes … they might be the people to actually advise’

Previous experience of the health‐care professional, as a subtheme, had both positive and negative attributes.

Nurse ‘there’s no point in talking about it to the patients if you can’t …give them some proper idea of how to actually carry on with what you’re talking about’

Physiotherapist ‘so I think if you’re in, into physical activity… yourself you’re going to have personal experience of it all and so be more engaged’

Another subtheme was that of the specific issues of the pathology of HD in people. Involuntary movements and poor balance were conveyed as both HD specific issues and a barrier to physical activity.

Nurse ‘their movements…. They don’t like them standing out’

Medical consultant ‘So would I feel comfortable telling a patient who’s kind of falling all over the place that they need to go out and do tai chi?’

Participants also recognized that a tool such as PAT‐HD facilitates consideration of specific HD issues.

Physiotherapist ‘there’s a real place for the pathway … supporting them to think about being physically active’

This relates closely to concepts within the key theme of *Approach*, that is how physical activity could be promoted.

*Approach* had two subthemes: individualized care and the use of PAT‐HD as an approach to promote physical activity. The need for an individualized approach was discussed in relation to the patients seen in clinic being at different stages of the condition, and therefore, different prompts and suggestions for activities were needed.

Nurse ‘it’s making it fall within every person’s ability’

Participants perceived that PAT‐HD prompted clinicians to initiate a conversation about physical activity, encouraged patients to think about physical activity and patients then had the completed hard copy of PAT‐HD to take home with them as a reminder.

Physiotherapist ‘I think it made people think about being more physically active… it’s good to have a prompt and something for them to take away’

Participants did provide feedback on PAT‐HD as a specific approach to promotion of physical activity. One participant felt that providing examples of activities, for example kick boxing, could be detrimental to the conversation with the person with HD.

Nurse ‘I think the other thing is the limitations of saying to people about, erm, things that they’re not capable of doing, that can be quite upsetting’.

Consequently, the list of activities on the front page of PAT‐HD was removed and replaced with illustrations depicting fitness, strength, flexibility and balance. The use of mobile phone applications was also highlighted as an approach to physical activity promotion by the participants.

Nurse ‘I know some of the clinical trials … are using apps and using special smart phones’

Maintaining physical activity engagement is a challenge for people with HD, and the approach of only using PAT‐HD in the clinic environment was seen as potentially problematic. Additionally when away from the support of the clinic remembering to use PAT‐HD, apathy, and implications for physical activity engagement as abilities change over time were perceived as challenges.

Nurse ‘they might say yes they’re quite capable of doing this and actually you realise they’re not…..they get home, they do it for a couple of days, and then they forget’

Within the key theme of *Setting* ***,*** two subthemes of clinic and community were identified. Time and clinic resources were salient issues, where lack of time and staff specifically dedicated to PAT‐HD meant that using PAT‐HD to promote general physical activity was overlooked due to other competing priorities such as research assessments for funded studies.

Physiotherapist ‘the time we’ve got available in the clinic, er, did limit that slightly’.

Nurse ‘I’m aware that I have to earn keep…. I think the clinic is quite a good, if it was funded or staffed properly’

The interviews elicited perceptions around the use of PAT‐HD in the community. Barriers to promoting physical activity in the community such as lack of local information and geographical spread of patients were discussed. However, perceived benefits to engaging people with HD within their local community included reduced social isolation and potential sustained engagement.

Medical consultant ‘…in the community definitely…’

Nurse ‘Even if it’s a walking group … that would be fantastic because you’re engaging within their community’

Physiotherapist ‘I think with someone that’s got allocated time …along with a resource pack for the local area’

## 4 DISCUSSION

We have described for the first time the co‐design and initial acceptability of a HD specific physical activity tool (PAT‐HD). PAT‐HD was co‐designed by people with HD, their families and carers and health‐care professionals with expertise in HD and acceptability was evaluated by health‐care professionals within a HD specific setting.

### 4.1 Phase 1: Development of a prototype physical activity tool

The prototype tool was developed using existing evidence in HD^6^, ^8^, ^11^ including a systematic review,^21^ recognized approaches to prescription of exercise^22^ and visual methodologies. Visual methodologies, for example use of illustrations, drawings and tables, can be used as a tool to collect research data and are considered as bridges between academic scholarship and applied public research^28^ and were therefore appropriate in the context of this study.

### 4.2 Phase 2*:* Co‐design of a tool to promote physical activity for people with HD

People with HD have limited experience of being supported to develop individualized physical activity plans.^29^ In order to facilitate interventions that support long‐term behavioural change in relation to physical activity, interventions must be developed by a range of stakeholders so as to be ecologically valid.^18^ The co‐design of PAT‐HD aligned with relevant principles of working in equal partnership for patient and public involvement^30^ and reduced perceived hierarchy^31^ by ensuring decision making was in the hands of the HD community rather than the researchers.

The complexity associated with the specific disease pathology in HD makes the planning and organization of co‐production activities more challenging.^32^ Typical barriers to co‐production include inadequate communication between stakeholders, time constraints and preconceptions about patients’ limitations to co‐produce.^33^ The involvement of a patient focussed organization to recruit specialist advisors and HD families enabled meaningful communication regarding design of the physical activity tool and use of a specialist facilitator at the event ensured equality in the voices heard.

### 4.3 Phase 3: Acceptability of the physical activity tool with health‐care professionals working in HD

Thematic analysis of the interviews with HD clinic staff using PAT‐HD identified three key themes of *People*, *Setting* and *Approach* with the theme of benefits and barriers being a continuous thread through each.

The theme of *People* included both the health‐care professional initiating a conversation about physical activity using PAT‐HD and the person with HD developing their physical activity plan. The participants, although acknowledging that they could all discuss physical activity with people with HD, felt that each professional had their own niche in which they worked and promotion of physical activity sat within the domain of physiotherapy in a HD clinic.

It has been recognized that physiotherapists have a unique position to promote healthy lifestyles based on a 100 year tradition of exercise prescription.^34^ Exercise prescription lends itself to a biomedical approach and subsequent work highlights a change towards a social ecological approach.^35^ Mulligan et al^35^ identified that physiotherapists working with people with neurological disability perceived a change in practice from one of a physiotherapist providing expert advice to resolve people's problems to an approach that supported people to attain independence and maintain physical activity. This aligns with a co‐production approach to health care that values health‐care professionals and patients co‐assessing, co‐deciding, co‐designing and co‐delivering care.^36^ PAT‐HD could therefore be used as an intervention to facilitate a co‐productive approach for people with HD to setting and achieving goals related to physical activity.

One participant felt that the degree to which physiotherapists could effectively promote physical activity depended upon their personal experience. There is limited evidence of relationships between personal physical activity levels and promotion of physical activity with other factors such as years of practice and length of consultation time being likely to influence practice in nurses.^37^

Specific consideration of HD issues was identified as a barrier to the promotion of physical activity by participants in the current study. One participant felt that carers did not want their family member to ‘stand out’ because of the choreic movements. Stigmatization associated with HD is well known^38^ with social stigma and lack of general public awareness of HD being perceived as a barrier to recruitment to a walking programme for people with HD.^32^ Altered balance may lead to a risk averse approach to promotion of physical activity, with one participant concerned about the likelihood of falls. Likelihood of falls should not be a barrier to activity, a risk benefit analysis of falls vs inactivity should guide practice.^12^

The study *Setting* was a HD specific clinic and was seen as advantageous by the participants as the person with HD was focused on their condition and its management during their clinic visit. Physical activity was only discussed with approximately half of those who attended the clinic; reasons given were advanced stage of the condition, severe mental health issues and lack of time. Insufficient time, which was already acknowledged by participants as a barrier to initiating a conversation on physical activity, was also identified as a barrier to developing an appropriate plan. Lack of time is a frequently cited barrier to promotion of physical activity, and this could be related to the range of tasks to be delivered by health‐care professionals within the consultation time period.^13^

Participants suggested that using PAT‐HD during an annual clinic visit was appropriate, yet they acknowledged this may not actually promote sustained behaviour change, partially due to apathy associated with the condition. Participants noted people with HD may exaggerate their capabilities leading to physical activity plans that cannot be achieved. Change in behaviour has three essential conditions: capability, opportunity and motivation,^39^ and therefore, a mismatch between perceived and actual capability may impact on successful completion of goals. Previous studies have demonstrated the feasibility of a HD physical activity self‐management intervention to be feasible, but did not include a long follow‐up period.^8^ Further work is needed to explore how increasing or maintaining physical activity levels can be sustained over the life span of HD.

Lack of resources is cited as a barrier to promotion of physical activity.^13^ PAT‐HD and the associated user guide were considered as resources and knowledge of appropriate local activities. Participants described examples of activities such as walking and activity groups but lacked knowledge of specific activities local to the person with HD. Time to identify these local opportunities was noted as a barrier. Settings such as a specific HD clinic or primary/community care were acknowledged as appropriate for promotion of physical activity. All participants suggested that promotion of physical activity may be better achieved in a community setting with increased local knowledge and discussions within the home embedding change within day‐to‐day life.

The theme of *Approach* included experience of using PAT‐HD as a tool and the importance of an individualized approach to physical activity promotion. Participants felt that PAT‐HD did support individualized care, taking into consideration the physical, cognitive and behavioural changes at different stages of the condition. Perceived benefits of this specific paper‐based tool were that it served as a focus for the person with HD to think about physical activity and as a visual reminder for when they returned to their home. There is limited evidence suggesting that patient information leaflets improve knowledge and change behaviour but, if used, the design should encourage patient interaction, be simple in presentation and have a structured layout.^40^ The co‐design of PAT‐HD ensured that the language, style and format of the tool was appropriate for people across the stages of the condition and the illustrations used simplified presentation.

### 4.4 Strengths and limitations

The strength of this study is the authenticity of PAT‐HD as a co‐designed intervention to promote physical activity for people with HD. The inclusion of just one HD clinic in the evaluation of PAT‐HD limits generalizability to other clinic settings; therefore, the study findings are based on initial acceptability.

### 4.5 Clinical implications

Development of this simple tool provides evidence that collaborative projects can be successfully undertaken with people with HD. PAT‐HD has potential to be used within HD specific clinics and in the community to co‐produce physical activity goals. There is also scope for the intervention to be developed for other conditions where physical activity is influenced by disease pathology.

### 4.6 Future work

Further work on the acceptability and efficacy of the tool with people with HD is needed, including assessment of maintenance of behaviour changes. We are currently living through the COVID‐19 pandemic which has raised challenges to the ways in which all community living individuals would normally engage in regular physical activity. A changing emphasis of keeping active within the home is needed, which aligns with the design of the front page of PAT‐HD with its focus on and distinction between moving more at home and moving more outside. Further work is, however, needed to develop systems to co‐ordinate up to date information on physical activity in localities and make this information available to health‐care professionals, when social distancing requirements become relaxed.

In an increasing technologically adept population, participants suggested mobile phone applications as an alternative approach to the paper‐based tool. Online professional‐patient interactions may be perceived to have challenges including information overload, ethics and data protection, but the benefits of allowing people time to think of questions relevant to them and development of a sense of community^41^ may be appropriate for people with HD. With social distancing potentially being in place for many months to come, use of PAT‐HD via tele video‐conferencing may be a feasible way to promote physical activity within the home.

## 5 CONCLUSION

Exercise interventions for people with HD have been demonstrated to be feasible, safe and effective; yet, the impact of research interventions on lives of people with HD is unknown. Health‐care professionals are in a position to promote physical activity during interactions, but evidence of this actually happening is limited with providing advice on exercise being perceived as challenging. A different approach to this biomedical model of diagnosis and resolution of problem/s and use of a social ecological approach to individualized care may be more appropriate for people with complex neurodegenerative conditions. Developing an understanding of a person's issues and perspectives within their locality may enhance shared decision making and therefore reduce the perceived responsibility of the health‐care professional to provide all the answers.

This study has demonstrated that a simple tool can be used to facilitate a conversation about physical activity with people with HD and to develop an individualized plan. Although a HD clinic may be an appropriate place to have this conversation, infrequency of clinical appointments means that continuity of care and re‐evaluation of the plan may not be sufficient to embed long‐term behaviour change. A community‐based approach that could include telehealth needs further exploration for people with HD.

## CONFLICT OF INTEREST

The authors declare that there is no conflict of interest.

## AUTHOR CONTRIBUTIONS

All authors designed the study. UJ collected the data. UJ and KH analysed the data. UJ, KH and MB participated in writing the paper.

## Data Availability

The data that support the findings of this study are available on request from the corresponding author. The data are not publicly available due to privacy or ethical restrictions.
